# *In vitro* evaluation of membranes for regenerative procedures against oral bacteria

**DOI:** 10.1590/0103-6440202305060

**Published:** 2023-07-17

**Authors:** Ana Clara Kuerten Gil, Maick Meneguzzo Prado, Laura Rhoden da Rocha, César Benfatti, Guenther Schuldt, Josiane de Almeida

**Affiliations:** 1 Department of Implant Dentistry, Federal University of Santa Catarina (UFSC), Florianópolis, Santa Catarina, Brazil; 2 Department of Chemical Engineering, Federal University of Santa Catarina (UFSC), Florianópolis, Santa Catarina, Brazil; 3 Department of Endodontics, University of Southern Santa Catarina (UNISUL), Palhoça, Santa Catarina, Brazil; 4 Department of Implant Dentistry, University of Southern Santa Catarina (UNISUL), Palhoça, Santa Catarina, Brazil

**Keywords:** bacteria, biofilm, biological membranes, guided bone regeneration, guided tissue regeneration, PTFE, collagen

## Abstract

The current literature on guided bone regeneration (GBR) and guided tissue regeneration (GTR) membrane contamination reports that the physicochemical characteristics of these biomaterials might influence affinity to bacteria, which appears to be a major drawback for the clinical outcome of the regenerative procedures. Thus, this study aimed to evaluate, in vitro, a multispecies biofilm adherence and passage of bacteria through different types of commercially available membranes for GTR/GBR. Four types of membranes were tested (n=12): LC) Lumina Coat®; JS) Jason®; BG) Biogide®; and LP) Lumina PTFE®. Aluminum foil (AL) simulated an impermeable barrier and was used as the control. The membranes were adapted to specific apparatus and challenged with a mixed bacterial culture composed of A. actinomycetemcomitans b, S. mutans, S. mitis, and A. israelii. After 2 h or 7 days, bacterial adhesion and passage of bacteria were evaluated through CFU counting, which was analyzed by two-way ANOVA e post hoc Tukey, at a 5% significance level. Representative areas of two membranes of each group were analyzed through scanning electron microscopy (SEM) to assess the morphology and organization of the biofilm over the membrane fibers. LC and LP presented similar values of adhered bacterial cells (p > 0.05), significantly inferior when compared to the other groups, in both time points (p < 0.05). All the tested groups were permeable to bacterial cells, with no significant difference between the trial period of 2 h and 7 days (p > 0.05). SEM analyses demonstrated that adhered bacteria number increased throughout the time points (2 h < 7 days). Commercially available biological membranes demonstrated intense bacterial adherence and passage of bacteria, which increased throughout the trial period.

## Resumo

O objetivo deste estudo foi avaliar, *in vitro*, a aderência do biofilme multiespécie e a passagem de bactérias através dos diferentes tipos de membranas disponíveis comercialmente para RTG/ROG. Quatro tipos de membranas foram testados (n=12): LC) Lumina Coat^®^; JS) Jason^®^; BG) Biogide^®^; e LP) Lumina PTFE^®^. Papel alumínio (AL) simulou uma barreira impermeável e foi usado como controle negativo. As membranas foram adaptadas à um aparato específico e desafiadas com uma cultura bacteriana mista composta de *A. actinomycetemcomitans b, S. mutans*, *S. mitis,* e *A. israelii.* Após 2 h ou 7 dias, a aderência e passagem bacteriana foi avaliada através da contagem de UFCs. Duas membranas de cada grupo foram analisadas através da microscopia eletrônica de varredura (MEV). LC e LP apresentaram valores semelhantes de células bacterianas aderidas (p < 0.05), significativamente inferiores quando comparados aos outros grupos, em ambos os períodos experimentais (p < 0.05). Desde a análise inicial, todos os grupos testados foram permeáveis às células bacterianas, sem diferença significativa entre o período experimental de 2 h e 7 dias (p > 0.05). As análises em MEV demonstraram que o número de bactérias aderidas aumentou com o tempo (2 h < 7 days). Membranas biológicas comercialmente disponíveis demonstraram intensa aderência bacteriana e passagem de bactérias, que aumentou durante os períodos experimentais.

## Introduction

The principles of guided tissue and bone regeneration (GTR/GBR) are based on the hindrance of epithelial and connective tissue cells in the rehabilitation area. The use of biological membranes in regenerative procedures protects blood clots and prevents the incursion of non-osteogenic cells into the surgical wound, providing an orderly bone repair ^(^ ^1^. 

The choice of membranes for GTR and GBR procedures usually depends on a professional preference aspect, yet there is still no agreement in the literature on which is the optimal biomaterial ^2^. The most used biological membranes are either resorbable, such as collagen ^3^ ^,^ ^4^, or non-resorbable, such as polytetrafluorethylene ^(^ ^5^. Clinically, both classes of biomaterials should remain entirely covered by gingival tissue to avoid microbial contamination ^6^ ^,^ ^7^. Resorbable membranes are permeable; thus, additional surgery for membrane removal is unnecessary. Therefore, regenerated bone exposure and patient morbidity are decreased. However, its rapid resorption process may harm bone formation ^(^ ^5^. Collagen-based biomaterials properties include biocompatibility, chemotaxis for osteoblasts and osteoblasts, hemostatic action, and semipermeable structure allowing the passage of nutrients ^3^ ^,^ ^4^. Although this biomaterial is considered the gold standard in GBR and largely used in dentistry ^3^, it exhibits fast biodegradation, at times influenced by microbial enzymatic activity, affecting membrane function ^5^ ^,^ ^8^ ^,^ ^9^. Hence, the seek for a resorbable membrane that remains in the body for a longer time evading bacterial degradation is shown in recent data of cellulose membranes research, in which inflammation and toxicity are not concerns despite the enhancement of degradation time ^3^. Also, biodegradable synthetic polymers such as polycaprolactone have drawn attention in this aspect, as polymers are generally degraded by hydrolysis whereas natural polymers such as collagen are mainly degraded enzymatically ^4^. Although non-resorbable membranes need additional surgery for removal, they still have advantages. For instance, dense polytetrafluorethylene (dPTFE) has low porosity and a high-density structure, hindering microbial invasion on the regeneration site. Hence, its impermeability grants the possibility of remaining exposed to the oral environment with reduced chances of contamination by exhibiting a barrier effect ^10^.

Failures in regenerative procedures are usually due to membranes becoming exposed to the oral environment ^11^ ^,^ ^12^ ^,^ ^13^, which can cause local inflammation, bone resorption, and ultimately early membrane retrieval ^11^ ^,^ ^14^. In these cases, healing occurs with granulation tissue instead of bone, impairing the treatment process ^14^. A significantly reduced mean regeneration rate was observed when membranes became prematurely exposed ^11^. 

Membrane properties such as chemical composition, surface energy, and roughness seem to benefit biofilm formation and passage of bacteria through its structure ^12^ ^,^ ^15^ ^,^ ^16^ ^)^ in the first hours ^15^ ^,^ ^16^ ^,^ ^17^ ^,^ ^18^. Various Gram-positive periodontal pathogens ^17^, such as *Streptococcus mutans* ^15^ ^,^ ^16^ ^,^ ^17^, *Actinobacillus actinomycetemcomitans* ^16^ ^,^ ^17^, *Porphyromonas gingivalis* ^18^, *Fusobacterium nucleatum*, *Actinomyces viscosus*, *Selenomonas sputigena* ^16^ are related to GTR/GBR contamination. These bacteria demonstrated the ability of adhesion and penetration in both resorbable and non-resorbable membranes ^15^ ^,^ ^16^ ^,^ ^17^ ^,^ ^18^ ^,^ ^19^ ^,^ ^20^ ^,^ ^21^ ^)^ even with the topic and systemic antimicrobial association ^13^ ^,^ ^17^ ^,^ ^22^. Complex adherence features were noted among various bacterial species ^21^, affecting the membrane performance and, therefore, jeopardizing soft tissue and bone repair ^12^ ^,^ ^13^ ^,^ ^23^. In vivo data demonstrated that the time needed for the complete passage of bacteria through an expanded polytetrafluorethylene (ePTFE) membrane structure was 4 weeks ^(^ ^11^. Also, the number of adhered bacteria to the membrane compromised the clinical attachment gain in GTR ^16^.

As bacterial colonization can be considered a major drawback for regenerative therapies, its success relies on an infection-free healing process, where microbial colonization should be restrained from the regeneration site ^8^ ^,^ ^24^ ^,^ ^25^ ^,^ ^26^. Studies have demonstrated the use of membranes and barriers in association with systemic and topic antimicrobial agents, as well as the improvement of its properties incorporating other components to decrease bacterial contamination ^3^ ^,^ ^27^ ^,^ ^28^. However, there is still no agreement in the current literature on an antimicrobial protocol related to these types of procedures. Furthermore, some biomaterials lack *in vitro* trials despite already being clinically applied. Hence, this research aimed to evaluate, *in vitro*, a multispecies biofilm adhesion and passage of bacteria through various types of commercially available membranes for clinical regenerative procedures. Also, this study is the first one to investigate microbial adherence and passage through Criteria^®^ collagen membrane and d-PTFE barrier, a Brazilian biomaterial brand that is widely used for GBR and GTR. Therefore, it emphasizes the evidence on this topic for further biomaterial improvements and clinical orientations.

## Materials and methods

### Bacterial species and inoculum preparation

The facultative anaerobic bacterial species *Aggregatibacter actinomycetemcomitans b* (ATCC 29523), *Streptococcus mutans* (ATCC 25175), *Streptococcus mitis* (ATCC49456), and *Actinomyces israelii* (ATCC 12102) were grown at 37 °C on brain heart infusion (BHI) agar (KASVI, Curitiba, PR, Brazil), under aerobic conditions. Strains were transferred from plates to BHI broth (KASVI) and incubated for 1-2 days under the appropriate conditions. Before each assay, the Optical Density (OD) of each culture was corrected to OD_600_ ≈ 0.5. 

### Membranes and experimental groups

**Figure 1:**
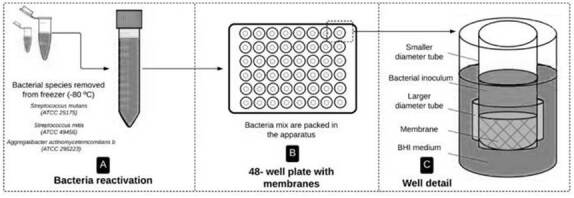
Schematic design from inoculum preparation to membrane disposition in transwell-like apparatus (A-C).

Different commercially available collagen and dPTFE membranes composed the experimental groups as follows: LC) Lumina Coat^®^ collagen membranes (Criteria, São Carlos, Brazil); JS) Jason^®^ collagen membranes (Straumann, Basel, Switzerland); BG) Bioguide^®^ collagen membranes (Geistlich Pharma, Zurich, Switzerland); LP) Lumina PTFE^®^ dense polytetrafluoroethylene membranes (Criteria, São Carlos, Brazil). Aluminum foil (AL) simulating barrier material was used as the negative control. Each membrane was adapted, individually and aseptically, into a transwell-shaped apparatus based on the study of Trobos et al. ^19^, formed by two sterile tubes; a smaller one with a 3 mm length and a bigger one with a 7 mm length. With an 8 mm inner diameter, the bigger one served as a docking base for the smaller tube (Ø = 7 mm) to fix the membrane in position and form a superior chamber for mixed bacterial culture placement (Figure 1).

### Biofilm formation on membranes

Firstly, 48-well culture plates were filled with 200 µL sterile BHI broth. Subsequently, the apparatus with the membranes (mobile good structure) were individually positioned inside each well so that the bottom section of the membranes maintain contact with the medium broth. The upper chamber of the wider length tubes was filled with 100 µL BHI medium of mixed culture (1:100 diluted; final concentration ≈ 10^6^ colony-forming unit (CFU)/mL) (supernatant) and incubated statically, at 37ºC, under anaerobic conditions, for 2 h and 7 days. During the experiment, the medium was refreshed after 2, 4, and 6 days.

### Bacterial adhesion analysis

To determine the number of attached viable cells on the membranes, after each time point was reached (2 h or 7 days), twelve apparatus per group were aseptically removed from the wells, the supernatant was discarded, and the membranes were collected with a sterile tweezer. Then, the membranes were rinsed with 0.9% sterile saline baths (3 × 1 mL) to remove non-adherent cells and transferred into tubes containing 1 mL of phosphate-buffered saline (PBS). The membranes were sonicated in an ultrasonic bath for 1 min at 40 kHz to dislodge the adherent bacterial cells and break bacterial aggregates. The bacterial suspensions obtained were vortexed at maximum speed (3200 rpm) for 1 min, and 100 µL of the suspensions were plated on BHI agar, in triplicate. Plates were incubated aerobically for 48 h, and the number of CFU/mL was determined. For that, the plates were divided into four quadrants, and the colony-forming units were spotted and automatically counted.

### Analysis of the biofilm structure

Two membranes per group of each experimental period were analyzed by scanning electron microscopy (SEM) (JEOL JSM-6390 LV; JEOL Ltd., Tokyo, Japan) to assess the biofilm architecture formed in the membrane surface ^28^. The membranes were rinsed with 0.9% sterile saline baths (3 × 1 mL) and fixed in 2.5% glutaraldehyde buffered with 0.2 M cacodylate at 4 °C for 12 h. After being washed with cacodylate buffer for 1 h and dehydrated with increasing grades of ethanol (25%, 50%, 75%, and 95% for 20 min for each concentration, and 100% for 1 h), they were dried via critical point drying method (EM CPD 030 / LEICA). The membranes were mounted on metallic stubs with the superior surface facing upwards and sputter-coated with a gold layer (300 Å). Two representative areas of each membrane were selected to assess the morphology and organization of the biofilm over the membrane fibers. The images were photographed from 300 × up to 3.000 × magnification, with the SEM operating the 10 kW. 

### Analysis of the passage of bacteria through the membranes

To assess the passage of the multispecies bacteria through the membranes, after apparatus removal from the wells, 100 µL of the culture medium (permeate) present on the lower chamber of each adapted transwell was collected and plated in BHI for CFU spot test. After 24 h, CFU/mL was determined as previously described. 

### Aseptic conditions of the experiment

To perform the experiment with no external contamination, all the commercial membranes used were acquired in the sterilized form. The aluminum foil, the transwell device, and all laboratory materials used were also autoclaved at 121ºC for 15 minutes previously to the experiments. The membranes were adapted to the apparatus on top of a sterilized surgical drape, using a sterilized tweezer. A Bunsen burner was used during the membrane installation to the device, once the hot air around the experiment decreases the chances of external contamination ^29^. All the stages regarding bacterial exposure were performed in the laminar flow under UV radiation, except for the UFC counting. The investigators were also vested with sterile gloves, disposable surgical caps, and masks during the whole experiment. 

### Statistical analysis

The data relating to CFU counting, obtained by adherence and permeability analyses of the different membranes, were analyzed by two-way ANOVA e *post hoc* Tukey, at a 5% significance level. The statistical analyses were performed through SPSS 21.0 software (IBM, Armonk, NY, USA).

## Results

### Bacterial adhesion

**Figure 2:**
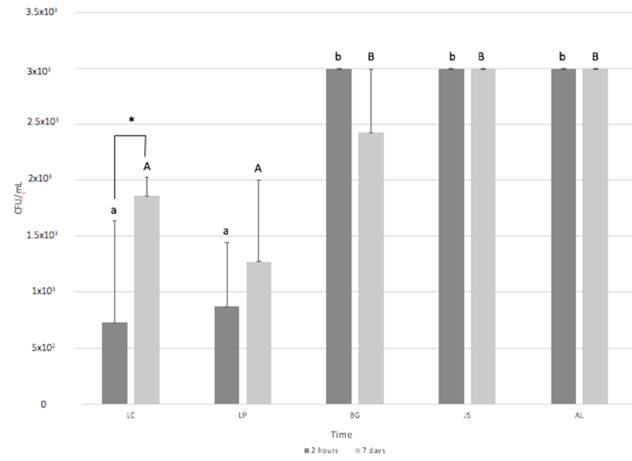
Bacterial adh erence (CFU/mL) to the experimental membranes after 2 hours and 7 days. *: significant difference between the periods of 2h and 7 days, within the same group. Different lowercase letters among the groups, in the period of 2h, indicate significant difference. Different capital letters among the groups, in the period of 2h, indicate significant difference.”

 A statistically significant increase in viable CFUs adhesion was only observed in LC collagen membranes when comparing the time points of 2 h and 7 days (p < 0.05). At 2 h, a lower adherence and biofilm formation was observed in LC and LP, with respectively 7.26 × 10^2^ and 8.73 × 10^2^ CFU/mL (p > 0.05), with a significant difference compared to the other groups (p < 0.05). At 7 days, the same pattern was noted related to adherence and biofilm formation. LC and LP presented similar values of adhered bacterial cells (p > 0.05), significantly lower when compared to the other groups (p < 0.05) (Figure 2). 

### Biofilm structure

**Figure 3:**
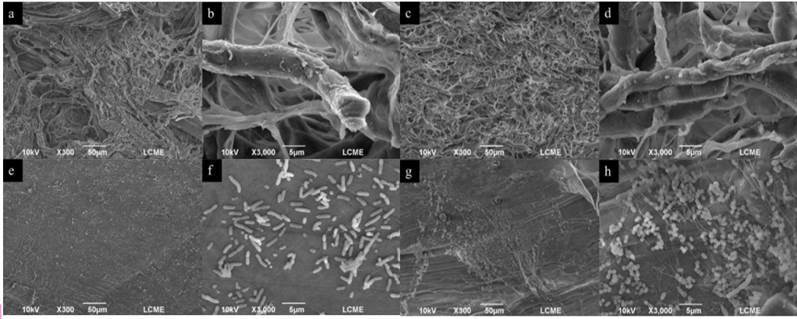
SEM images showed increased of bacterial adherence to LC and LP membranes during the experimental periods. LC - 2 hours (A-B); LC - 7 days (C-D); LP - 2 hours (E-F); LP - 7 days. (magnification ×300 and ×3,000). LC, Lumina Coat^®^; LP, Lumina PTFE^®^.

**Figure 4:**
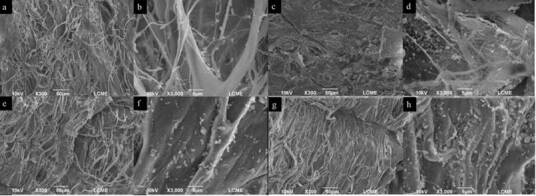
SEM images showed increased bacterial adherence to JS and BG membranes along the experimental periods. JS - 2 hours (A-B); JS - 7 days (C-D); BG - 2 hours (E-F); BG - 7 days. (magnification ×300 and ×3,000). JS, Jason^®^; BG, Bioguide^®^.

Qualitative analysis of biofilm through SEM demonstrated, in general, that the number of bacteria adhered to the membranes increased throughout the trial period (2 h < 7 days) (Figures 3 and 4). Few bacterial aggregates were observed distributed among the collagen fibers of LC membranes shortly after 2 h. However, at 7 days, some of the fibers demonstrated organized and adhered biofilm, mainly composed of *cocci*. Microorganisms, predominantly rod-shaped, spread isolated, or clustered, were present over the smooth surface of the LP membranes at the initial analysis (2 h). At 7 days, there was an increase of adhered bacteria, predominantly cocci, spread in clusters over the membrane surface. JS and BG membranes showed similar image patterns in both periods. Isolated bacteria adhered and interspersed in the collagen fibers were noted at 2 h, which evolved to dense bacterial clusters with an immature biofilm area at 7 days of analysis. 

### Passage of bacteria through the membranes

**Figure 5:**
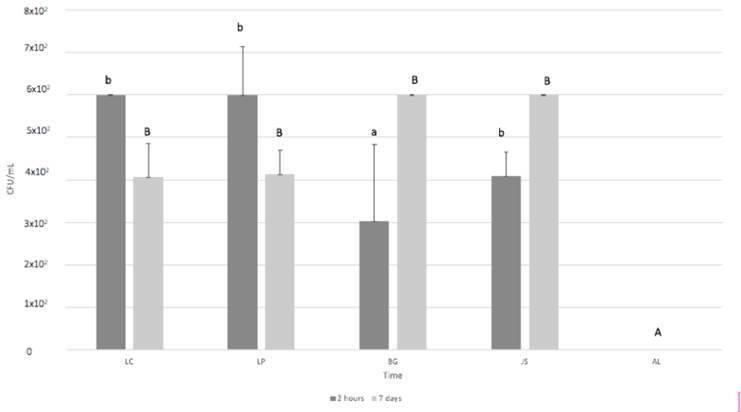
Viable bacterial penetration (CFU/mL) through the experimental membranes at 2 hours and 7 days. Different lowercase letters among the groups, in the period of 2h, indicate significant difference. Different capital letters among the groups, in the period of 2h, indicate significant difference.”

All the tested membranes allowed the passage of bacteria, with no significant difference between the time points of 2 h and 7 days (p > 0.05). None of the control group samples enabled the passage of bacteria in both time points (p > 0.05). BG had the most significant effect on preventing the passage of bacteria at 2 h compared to the other membranes (p < 0.05). However, at 7 days, there was no significant difference in CFU/mL counting among the experimental groups (p > 0.05), but higher values were observed compared to the control group (p < 0.05) (Figure 5). 

## Discussion

Membranes that stay completely covered by gingival tissue during the healing/regeneration process tend to remain protected from microorganism invasion, allowing adequate bone repair ^12^. On the other hand, membrane exposure to the oral environment may jeopardize the regeneration process and, consequently, the patient rehabilitation ^11^ ^,^ ^12^ ^,^ ^13^ ^,^ ^14^. Structural characteristics of such biomaterials might facilitate bacterial colonization, adhesion, and penetration ^12^ ^,^ ^15^ ^,^ ^16^. Therefore, this investigation aimed to evaluate bacterial adherence and the passage of bacteria through the various types of commercially available membranes for GTR/GBR clinical procedures. 

This study used aluminum foil as the control, simulating the barrier effect of a membrane. As expected, bacterial adherence to the material surface exposed to multispecies inoculum was identified, but there was no evidence of the passage of bacteria in both time points of 2 hours and 7 days. The choice of experimental periods was based on the biofilm structure organization, as at 2 hours is still an unorganized biofilm, while at 7 days a more structured and arranged biofilm, thus, it could be clearly distinguished the immature from the mature biofilm, and the respective predominant shapes of the microbes. 

Bacterial adherence to membranes throughout the trial period corroborates in vitro findings, that indicate greater adherence as the time of exposure to the oral cavity increases ^14^. Nevertheless, in the first two hours of the experiment, bacterial adherence was more pronounced in BG and JS groups. Since microbial adherence to biomaterials varies according to the structure ^16^ and substrate texture ^30^, the affinity to collagen membranes can be explained due to its low hydrophobicity. The more hydrophilic the material, the greater the propensity to bacterial adherence ^31^. Therefore, this type of membrane is recommended only when a complete soft tissue coverage of the rehabilitation area can be achieved ^25^. Bacterial adherence was significantly higher in BG and JS compared to LC. That may have occurred because of the nature and the distinct disposition of collagen fibers that compose the membranes. Such structural properties alter and promote different bacterial characteristics regarding adherence ability ^31^.

The lower number of microorganisms adhered to the LP membranes endorses previous studies (16-21). These biomaterials' dense and pore-free structure surpasses ePTFE microstructure disadvantages, whose porosities facilitate microbial adherence and penetration ^16^. Conversely, evidence revealed that the passage of bacteria through the ePTFE membranes could be hampered for 3 to 4 weeks due to its reduced porosity ^11^. However, previous literature still shows a divergence in conclusions relating to bacterial adhesion to membranes, particularly the dissimilarities between the dense PTFE structure, utilized in this research, and the ePTFE layout ^19^ ^,^ ^25^. Paradoxically, a previous investigation demonstrated a reduction of bacterial colonization as the surface porosity of PTFE increases ^19^. In contrast, another study exhibited no significant difference in bacterial adherence between ePTFE and dPTFE membranes ^(^ ^25^. Interestingly, although dPTFE is considered a barrier, impermeable to microorganisms, cells, and fluids ^10^, the passage of bacteria was evident in all trial periods of the present investigation. This is potentially due to the struggle for membrane adaptation to transwell-like apparatus, which may have provided false-positive results.

Although the data indicate that surface porosity and interstitial space among fibers are inversely proportional to the barrier effect ^18^, previous findings showed that resorbable membranes hold similar barrier effect potential compared to non-resorbable membranes ^25^, supporting the finding of the present study. It should be emphasized that permeability variation among the tested membranes may occur due to the size of the bacteria cells used in the experiments. For example, *S. mutans* diameter varies between 1 to 0.5 μm and *A. actynomycetemcomitans* from 1.0 to 1.5 × 0.4 to 0.5 μm. Membranes with larger pores could make the passage of bacteria viable ^16^ ^,^ ^17^. While BG membranes presented slightly inferior permeability at 2 hours, there was an increase of CFU/mL at 7 days, with no difference among the other groups. Collagen membranes are susceptible to premature degradation by proteolytic bacteria, which impairs a complete repair process ^8^ ^,^ ^14^ ^,^ ^25^. To avoid this, cross-linking methods for collagen membranes have been created through chemical and physical improvement ^5^ ^,^ ^31^.

Scanning electron microscopy is constantly applied in GTR/GBR membrane analyses. Such technology discloses the diverse bacterial adherence patterns, highlighting the membrane ability to attract microorganisms ^11^ ^,^ ^12^ ^,^ ^13^ ^,^ ^16^ ^,^ ^17^ ^,^ ^18^ ^,^ ^19^ ^,^ ^23^ ^,^ ^24^ ^,^ ^25^ ^,^ ^30^. Biofilm formation in biomaterials follows the same standards of plaque development in enamel, cementum, and titanium implants, where primary colonization is predominantly cocci ^30^, similar to most of the images from the present study (Figures 1 and 2). 

The research on GTR/GBR biomaterial contamination and its consequent failure is of utmost importance for a clinical approach that prevents and promotes solutions for this drawback. Thus, investigations that employ longer trial periods and a wider variety of bacterial species are of significant value. Such discoveries may achieve results closer to clinical reality and provide new biomaterial development, able to circumvent the obstacles of microbial infection. 

## Conclusion

It can be concluded that bacterial adherence and passage of bacteria through the membranes were evident in all tested groups, which increased throughout the trial period. From a clinical perspective, such findings require particular attention from clinicians on regenerative treatment planning, considering attainable ways of controlling microorganisms before, during, and after the procedure. In addition, further investigations that contemplate alternatives such as structural refinement of membranes are essential to enhance the understanding of biomaterial antimicrobial properties. 
